# Chlorido­[5,10,15,20-tetra­kis­(quinoline-7-carboxamido)­porphinato]iron(III)

**DOI:** 10.1107/S2414314624004966

**Published:** 2024-06-04

**Authors:** Jun Yang, Cuijuan Zhang, Jiaxiang Chu

**Affiliations:** a School of Chemical Sciences University of Chinese Academy of Sciences, 101408 Beijing People’s Republic of China; University of Aberdeen, United Kingdom

**Keywords:** crystal structure, iron, porphyrin derivative, hydrogen bonds

## Abstract

The title compound, [Fe(C_84_H_52_N_12_O_4_)Cl], crystallizes in space group *C*2*/c*. The central Fe^III^ cation (site symmetry 2) is coordinated in a fivefold manner, with four pyrrole N atoms of the porphyrin core in the basal sites and one Cl atom (site symmetry 2) in the apical position, which completes a slightly distorted square-pyramidal environment. The porphyrin macrocycle shows a characteristic ruffled-shape distortion and the iron atom is displaced out of the porphyrin plane by 0.42 Å with the average Fe—N distance being 2.054 (4) Å; the Fe—Cl bond length is 2.2042 (7) Å. Inter­molecular C—H⋯N and C—H⋯O hydrogen bonds occur in the crystal structure.

## Abstract 2

The porphyrin macrocycle shows a characteristic ruffled-shape distortion. The central Fe^III^ cation (site symmetry 2) is coordinated in a fivefold manner, with four pyrrole N atoms of the porphyrin core in the basal sites and one Cl atom (site symmetry 2) in the apical position.

## Graphical abstract

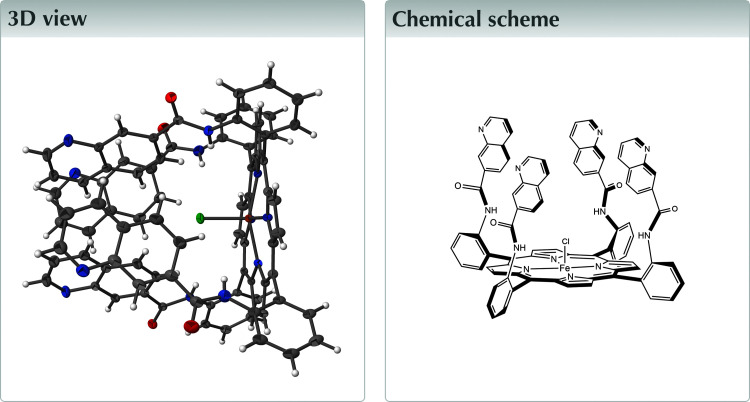

## Structure description

The relationship between the structural variations of iron porphyrins and the functional diversity of hemoproteins has been investigated extensively in the literature (Adam *et al.*, 2018 ▸). As an exemplar porphyrin model, the mol­ecular structure of the ‘picket-fence’ porphyrin, 5,10,15,20-tetra­kis­(*o*-pivalamido­phen­yl)porphyrin (referred to as TpivPP) has been thoroughly investigated. The Collman group first reported the crystal structure of a picket-fence metalloporphyrin, [Fe(TpivPP)(1-MeIm)(O_2_)] (1-MeIm = 1-methyl­imid­azole; Collman *et al.*, 1975 ▸). Subsequently, several analogues of picket-fence compounds have been synthesized. The Gunter group devised a model compound, 5,10,15,20-tetra­kis­(*o*-nicotinamido­phen­yl)porphyrin, modeled after the classical picket-fence porphyrin, with the substitution of the *tert*-butyl group at the terminus by a pyridine group (Gunter *et al.*, 1980 ▸). Similarly, Yao and co-workers developed 5,10,15,20-tetra­kis­(5-imidazole­carboxyl­amino­phen­yl)porphyrin by substituting the *tert*-butyl group at the terminus with an imidazole group (Yao *et al.*, 2020 ▸). In this study, we replaced the terminal *tert*-butyl group with a 7-quinoline group, and determined the crystal structure of the title compound [Fe(C_84_H_52_N_12_O_4_)Cl].

**Figure 1:**
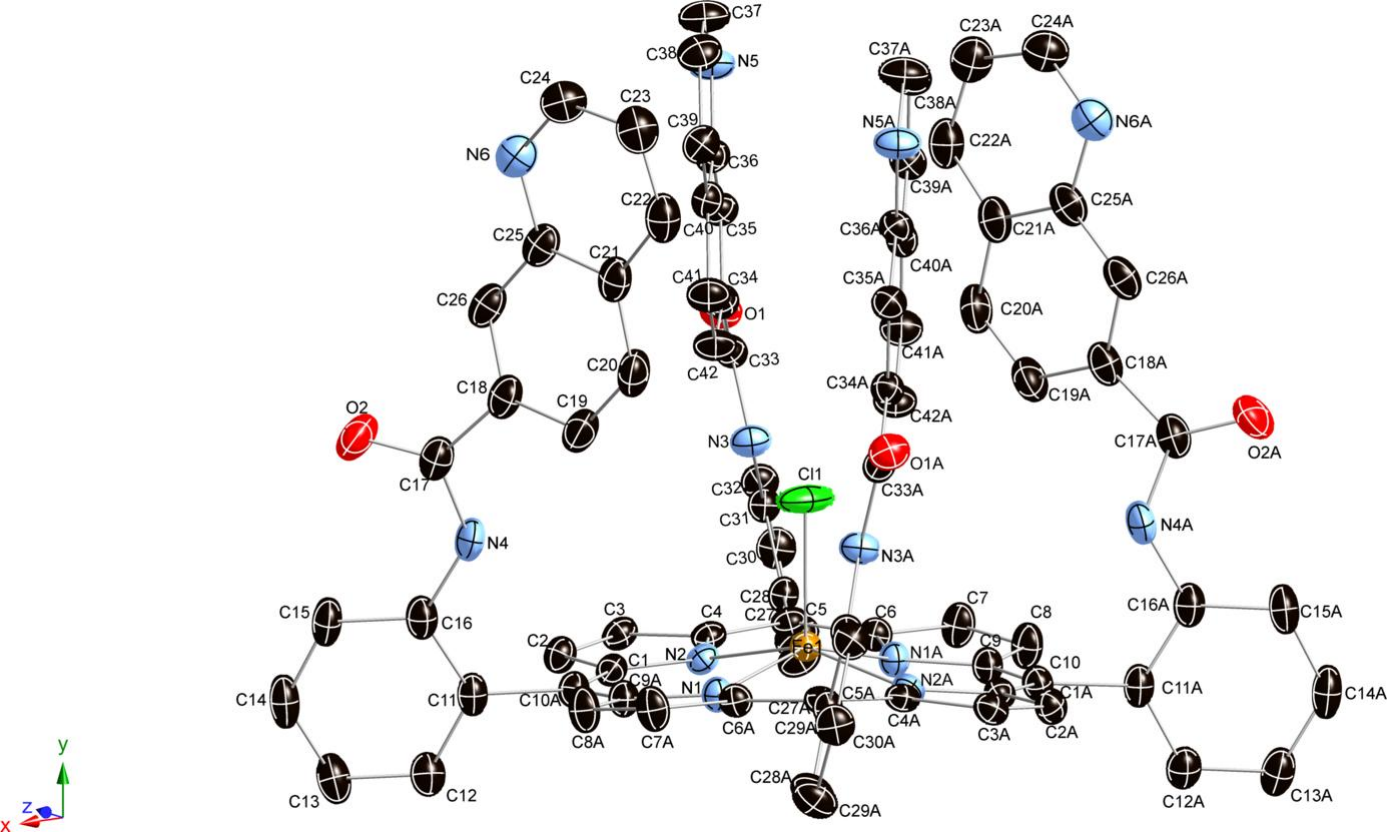
The mol­ecular structure of the title compound with displacement ellipsoids drawn at the 50% probability level. Hydrogen atoms have been omitted for clarity.

The asymmetric unit contains one Fe atom and one Cl atom (both site symmetry 2) and half of the porphyrin ligand, which is completed by crystallographic twofold symmetry. There are no solvent mol­ecules present in the crystal. As depicted in Fig. 1 ▸, the new crystal demonstrates a five-coordinate structure of the metal atom with a significant out-of-plane displacement. The axial chloride ligand is positioned within the mol­ecular cavity on the hindered porphyrin side. Further structural details are presented in supplementary Fig. 1 ▸, including the specific displacements of each porphyrin core atom from the 24-atom mean plane. Additionally, averaged values of the chemically unique bond lengths (Å) and angles (°) are provided. Notably, the porphyrin macrocycle exhibits a characteristic ruffled-shaped distortion, with the Fe^III^ atom displaced out of the porphyrin plane by 0.42 Å, and an average Fe—N_p_ distance of 2.054 (4) Å (N_p_ represents a porphyrin N atom). The Fe—Cl bond length is 2.2042 (7) Å (Table 1 ▸).

**Figure 2:**
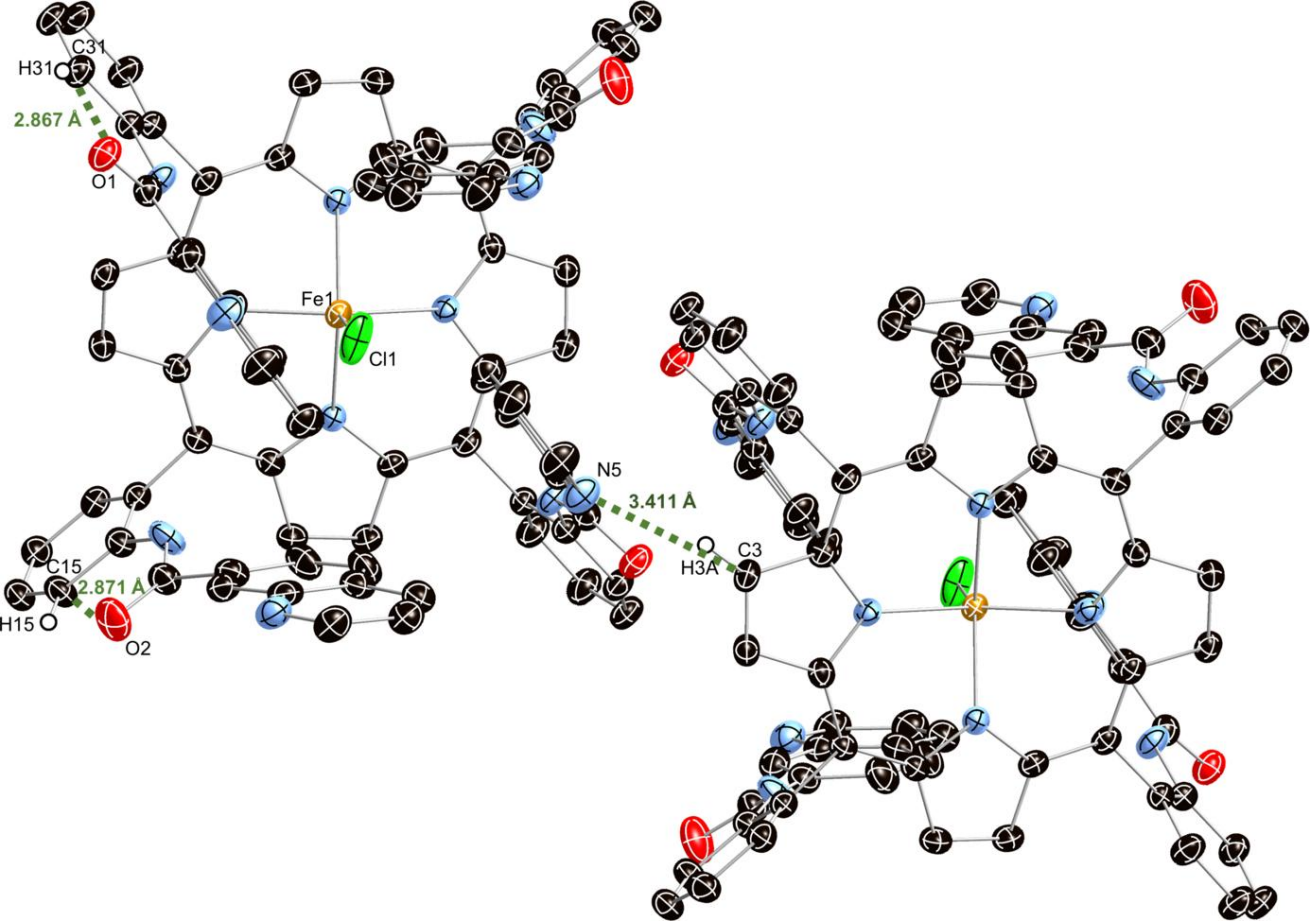
Intra- and inter-mol­ecular inter­actions in the crystal structure of the title compound.

**Figure 3:**
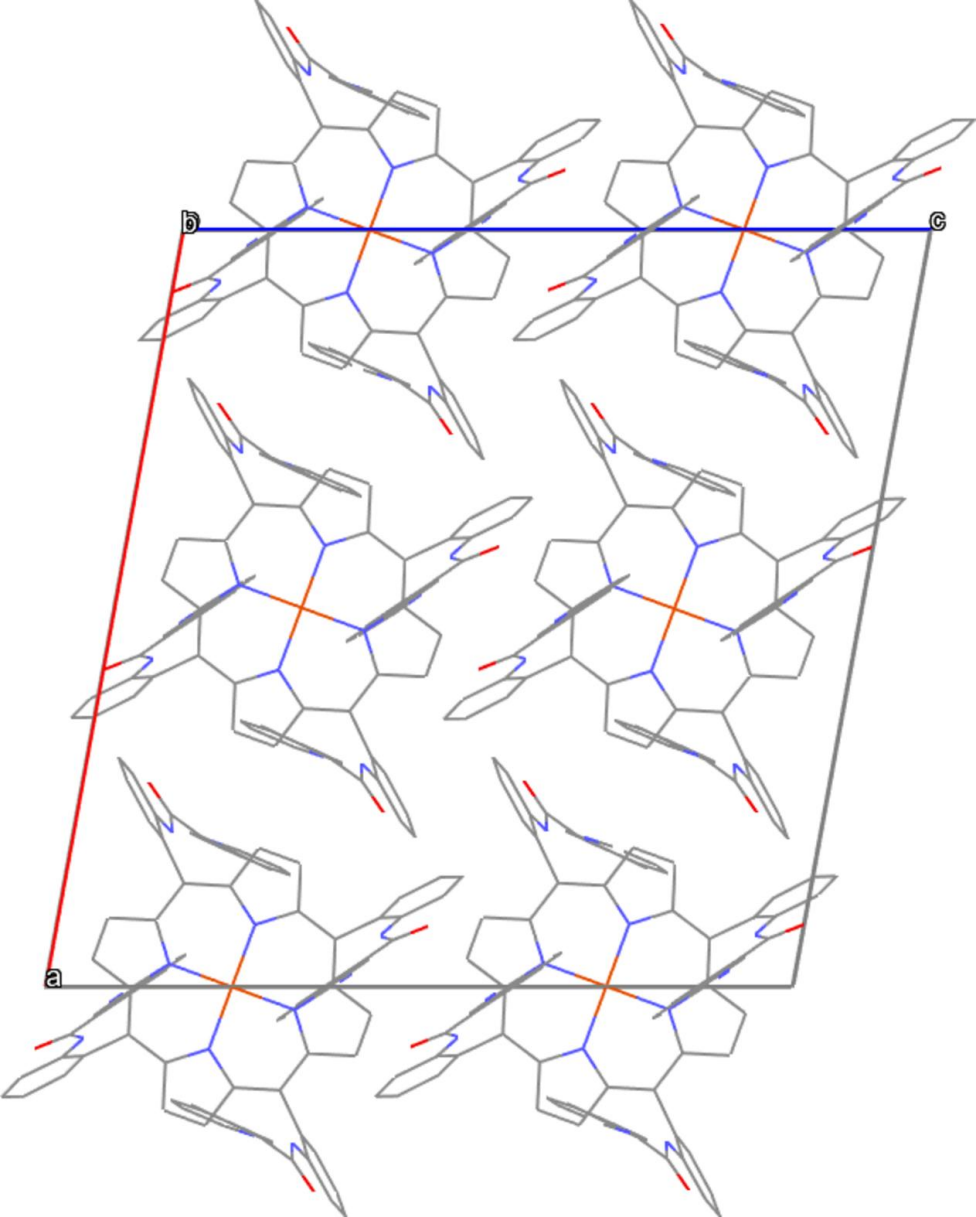
A view of the packing of the title compound. H atoms have been omitted for clarity.

Several intra- and inter-mol­ecular inter­actions are identified in the title compound, as presented in Table 2 ▸ and Fig. 2 ▸. The distance between C3 and N5 and the C3—H3*A*⋯N5 angle are 3.411 (2) Å and 166°, respectively. These value are consistent with literature data where the C⋯N separation of C—H⋯N hydrogen bonds ranges from 2.4–3.9 Å (Rabaça *et al.*, 2022 ▸) with angles of 100–171° (Shivakumar *et al.*, 2012 ▸). Furthermore, the distances between C15 and O2, and C31 and O1 are 2.871 (3) Å and 2.867 (2) Å, respectively, which align with literature data where the C⋯O separation of C—H⋯O bonds ranges from 3.00–4.00 Å (Desiraju, 1996 ▸) with angles of 120–180° (Thakur *et al.*, 2015 ▸). The mol­ecular packing arrangement is illustrated in Fig. 3 ▸.

## Synthesis and crystallization

All experimental procedures were carried out under an argon atmosphere using a double-manifold vacuum line, Schlenkware, and cannula techniques. Except for the solvent employed in column chromatography, all solvents used in the experimental protocols underwent thorough drying and purging under anhydrous and anaerobic conditions. Solvents utilized within the anhydrous and anaerobic operations (Schlenk system) underwent the freeze–pump–thaw method three times prior to utilization.

The synthesis of the precursor 5,10,15,20-tetra­kis­(quinoline-7-carboxamide)­porphyrin followed the procedures outlined in a previous publication (Yao *et al.*, 2020 ▸). Initially, oxalyl chloride (2.2 mmol) was added to a suspension of 7-quinoline­carb­oxy­lic acid (1 mmol) in a solvent mixture of 15 ml di­chloro­methane (DCM) and 10 µl *N*,*N*-di­methyl­formamide in a nitro­gen-protected atmosphere. The reaction mixture was stirred at room temperature for 1 h and concentrated *in vacuo*. The resulting solid was used in the subsequent step without further purification. Dry DCM (25 ml) containing 7-quinoline­carb­oxy­lic acid chloride was mixed with *αααα*-H_2_TamPP (0.2 mmol) and 2,6-lutidine (270 µmol). The resulting solution was refluxed for 90 minutes and concentrated to dryness. The obtained product was purified through chromatography on a silica gel column using an elution solvent mixture of CHCl_3_:CH_3_OH in a ratio of 12:1, resulting in a yield of 70%. Subsequently, the chloro-iron porphyrin compound was prepared. To a solution of the free base porphyrin (*ca*. 100 µmol) in tetra­hydro­furan (30 ml), FeCl_2_ (20 equiv) and 2,6-lutidine (50 µl) were added. The mixture was refluxed overnight and concentrated to dryness. The resulting product was purified through chromatography on a silica gel column using an elution solvent composed of CHCl_3_:CH_3_OH in a ratio of 9:1, resulting in a yield of 70%. To produce X-ray-quality crystals, we utilized a vapor diffusion technique, wherein *n*-hexane was introduced into a 3 m*M* di­chloro­methane (CH_2_Cl_2_) solution to initiate crystallization.

## Refinement

Crystal data, data collection and structure refinement details are summarized in Table 3 ▸.

## Supplementary Material

Crystal structure: contains datablock(s) I. DOI: 10.1107/S2414314624004966/hb4470sup1.cif

Structure factors: contains datablock(s) I. DOI: 10.1107/S2414314624004966/hb4470Isup2.hkl

Porphyrin ring displacement data. DOI: 10.1107/S2414314624004966/hb4470sup3.docx

CCDC reference: 2358334

Additional supporting information:  crystallographic information; 3D view; checkCIF report

## Figures and Tables

**Table 1:** Selected geometric parameters (Å, °)

Fe1—Cl1	2.2042 (7)	Fe1—N2	2.0581 (13)
Fe1—N1	2.0508 (13)		
			
N1—Fe1—Cl1	102.93 (4)	N2—Fe1—Cl1	101.67 (4)
N1^i^—Fe1—N1	154.14 (8)	N2^i^—Fe1—N2	156.66 (7)
N1—Fe1—N2^i^	87.60 (5)		

Symmetry code: (i) 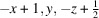.

**Table 2:** Hydrogen-bond geometry (Å, °)

*D*—H⋯*A*	*D*—H	H⋯*A*	*D*⋯*A*	*D*—H⋯*A*
C15—H15⋯O2	0.95	2.25	2.871 (3)	122
C31—H31⋯O1	0.95	2.25	2.867 (2)	122
C42—H42⋯Cl1	0.95	2.77	3.3596 (18)	121
C3—H3*A*⋯N5^ii^	0.95	2.48	3.411 (2)	166
C14—H14⋯O1^iii^	0.95	2.45	3.165 (2)	132

Symmetry codes: (ii) 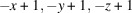; (iii) 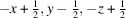.

**Table 3:** Experimental details

Crystal data	
Chemical formula	[Fe(C_84_H_52_N_12_O_4_)Cl]
*M*_r_	1384.68
Crystal system, space group	Monoclinic, *C*2/*c*
Temperature (K)	100
*a*, *b*, *c* (Å)	23.1771 (19), 12.7959 (10), 22.5404 (16)
β (°)	100.332 (3)
*V* (Å^3^)	6576.5 (9)
*Z*	4
Radiation type	Mo *K*α
μ (mm^−1^)	0.34
Crystal size (mm)	0.47 × 0.24 × 0.24

Data collection	
Diffractometer	Bruker APEXII CCD
Absorption correction	–
No. of measured, independent and observed [*I* > 2σ(*I*)] reflections	56518, 6685, 5979
*R*_int_	0.041
(sin θ/λ)_max_ (Å^−1^)	0.626

Refinement	
*R*[*F*^2^ > 2σ(*F*^2^)], *wR*(*F*^2^), *S*	0.035, 0.088, 1.07
No. of reflections	6685
No. of parameters	469
H-atom treatment	H atoms treated by a mixture of independent and constrained refinement
Δρ_max_, Δρ_min_ (e Å^−3^)	0.38, −0.46

Computer programs: *APEX2* and *SAINT* (Bruker, 2014 ▸), *OLEX2.solve* (Bourhis *et al.*, 2015 ▸), *OLEX2* (Dolomanov *et al.*, 2009 ▸) and *SHELXL2018/3* (Sheldrick, 2015 ▸).

## full crystallographic data

### Chlorido[5,10,15,20-tetrakis(quinoline-7-carboxamido)porphinato]iron(III) Crystal data

**Table:**

[Fe(C_84_H_52_N_12_O_4_)Cl]	*F*(000) = 2860
*M**_r_* = 1384.68	*D*_x_ = 1.399 Mg m^−^^3^
Monoclinic, *C*2/*c*	Mo *K*α radiation, λ = 0.71073 Å
*a* = 23.1771 (19) Å	Cell parameters from 9906 reflections
*b* = 12.7959 (10) Å	θ = 2.5–26.4°
*c* = 22.5404 (16) Å	µ = 0.34 mm^−^^1^
β = 100.332 (3)°	*T* = 100 K
*V* = 6576.5 (9) Å^3^	Block, black
*Z* = 4	0.47 × 0.24 × 0.24 mm

### Chlorido[5,10,15,20-tetrakis(quinoline-7-carboxamido)porphinato]iron(III) Data collection

**Table:**

Bruker APEXII CCD diffractometer	*R*_int_ = 0.041
φ and ω scans	θ_max_ = 26.4°, θ_min_ = 2.3°
56518 measured reflections	*h* = −28→28
6685 independent reflections	*k* = −16→16
5979 reflections with *I* > 2σ(*I*)	*l* = −28→25

### Chlorido[5,10,15,20-tetrakis(quinoline-7-carboxamido)porphinato]iron(III) Refinement

**Table:**

Refinement on *F*^2^	Primary atom site location: iterative
Least-squares matrix: full	Hydrogen site location: mixed
*R*[*F*^2^ > 2σ(*F*^2^)] = 0.035	H atoms treated by a mixture of independent and constrained refinement
*wR*(*F*^2^) = 0.088	*w* = 1/[σ^2^(*F*_o_^2^) + (0.0246*P*)^2^ + 12.3743*P*] where *P* = (*F*_o_^2^ + 2*F*_c_^2^)/3
*S* = 1.07	(Δ/σ)_max_ = 0.001
6685 reflections	Δρ_max_ = 0.38 e Å^−^^3^
469 parameters	Δρ_min_ = −0.46 e Å^−^^3^
0 restraints	

### Chlorido[5,10,15,20-tetrakis(quinoline-7-carboxamido)porphinato]iron(III) Special details

**Table:**

Geometry. All esds (except the esd in the dihedral angle between two l.s. planes) are estimated using the full covariance matrix. The cell esds are taken into account individually in the estimation of esds in distances, angles and torsion angles; correlations between esds in cell parameters are only used when they are defined by crystal symmetry. An approximate (isotropic) treatment of cell esds is used for estimating esds involving l.s. planes.

### Chlorido[5,10,15,20-tetrakis(quinoline-7-carboxamido)porphinato]iron(III) Fractional atomic coordinates and isotropic or equivalent isotropic displacement parameters (Å^2^)

**Table:**

	*x*	*y*	*z*	*U*_iso_*/*U*_eq_	
Fe1	0.500000	0.19602 (2)	0.250000	0.01406 (8)	
Cl1	0.500000	0.36828 (5)	0.250000	0.03714 (17)	
O1	0.41885 (5)	0.50579 (9)	0.49890 (5)	0.0241 (3)	
N1	0.41780 (6)	0.16016 (10)	0.26565 (6)	0.0170 (3)	
N3	0.43287 (6)	0.37351 (11)	0.43536 (6)	0.0205 (3)	
N2	0.53043 (5)	0.16349 (10)	0.33957 (6)	0.0150 (3)	
O2	0.22888 (6)	0.45237 (12)	0.08918 (6)	0.0395 (3)	
N6	0.30417 (7)	0.79208 (13)	0.18249 (7)	0.0305 (3)	
N5	0.48009 (7)	0.83155 (12)	0.40947 (7)	0.0280 (3)	
N4	0.29534 (7)	0.33119 (13)	0.13238 (7)	0.0283 (3)	
C5	0.43699 (7)	0.17883 (12)	0.37670 (7)	0.0161 (3)	
C27	0.40879 (7)	0.19029 (13)	0.43123 (7)	0.0170 (3)	
C4	0.49801 (7)	0.17192 (12)	0.38512 (7)	0.0157 (3)	
C33	0.43741 (7)	0.47455 (13)	0.45470 (7)	0.0189 (3)	
C3	0.53593 (7)	0.16678 (12)	0.44304 (7)	0.0180 (3)	
H3A	0.524266	0.170362	0.481303	0.022*	
C6	0.39996 (7)	0.17128 (12)	0.32058 (7)	0.0173 (3)	
C9	0.36683 (7)	0.15418 (13)	0.22305 (7)	0.0189 (3)	
C40	0.51885 (7)	0.69603 (13)	0.35087 (7)	0.0202 (3)	
C32	0.40714 (7)	0.28739 (13)	0.46001 (7)	0.0179 (3)	
C36	0.48723 (7)	0.72863 (13)	0.39614 (7)	0.0198 (3)	
C1	0.58807 (7)	0.15469 (12)	0.36826 (7)	0.0165 (3)	
C10	0.36335 (7)	0.14926 (13)	0.16065 (7)	0.0182 (3)	
C11	0.30400 (7)	0.14485 (14)	0.12114 (7)	0.0209 (3)	
C39	0.54339 (8)	0.77432 (14)	0.31872 (8)	0.0240 (4)	
H39	0.564563	0.755848	0.287869	0.029*	
C2	0.59132 (7)	0.15590 (13)	0.43272 (7)	0.0197 (3)	
H2	0.625887	0.150127	0.462395	0.024*	
C35	0.46154 (7)	0.65284 (13)	0.42850 (7)	0.0198 (3)	
H35	0.440172	0.674535	0.458579	0.024*	
C34	0.46684 (7)	0.54836 (13)	0.41734 (7)	0.0191 (3)	
C28	0.38285 (8)	0.10387 (14)	0.45276 (8)	0.0243 (4)	
H28	0.384045	0.038161	0.433394	0.029*	
C18	0.30038 (7)	0.50730 (15)	0.17230 (8)	0.0266 (4)	
C31	0.38000 (7)	0.29535 (14)	0.51067 (7)	0.0219 (3)	
H31	0.379183	0.360378	0.530841	0.026*	
C13	0.22732 (8)	0.04515 (16)	0.05918 (8)	0.0301 (4)	
H13	0.211626	−0.020147	0.044043	0.036*	
C8	0.31670 (7)	0.15940 (15)	0.25228 (8)	0.0250 (4)	
H8	0.276745	0.154920	0.233226	0.030*	
C26	0.29544 (7)	0.61104 (15)	0.15667 (8)	0.0256 (4)	
H26	0.279465	0.630038	0.116320	0.031*	
C41	0.52447 (8)	0.58790 (14)	0.34060 (8)	0.0252 (4)	
H41	0.545774	0.564905	0.310748	0.030*	
C25	0.31377 (7)	0.68972 (15)	0.19966 (8)	0.0254 (4)	
C12	0.28099 (7)	0.04982 (15)	0.09838 (7)	0.0250 (4)	
H12	0.302142	−0.012795	0.109711	0.030*	
C38	0.53627 (8)	0.87648 (14)	0.33257 (8)	0.0286 (4)	
H38	0.552389	0.930464	0.311643	0.034*	
C29	0.35514 (8)	0.11235 (15)	0.50233 (8)	0.0280 (4)	
H29	0.336836	0.053119	0.516364	0.034*	
C30	0.35440 (8)	0.20768 (14)	0.53110 (8)	0.0253 (4)	
H30	0.336037	0.213161	0.565423	0.030*	
C21	0.33789 (7)	0.66073 (15)	0.25996 (8)	0.0273 (4)	
C7	0.33700 (7)	0.17192 (15)	0.31210 (8)	0.0245 (4)	
H7	0.313943	0.179663	0.342672	0.029*	
C16	0.27200 (7)	0.23702 (15)	0.10545 (8)	0.0240 (4)	
C15	0.21837 (7)	0.23219 (16)	0.06521 (8)	0.0282 (4)	
H15	0.196822	0.294312	0.053655	0.034*	
C24	0.31731 (9)	0.86345 (17)	0.22469 (9)	0.0350 (4)	
H24	0.310255	0.934447	0.213352	0.042*	
C42	0.49958 (8)	0.51593 (14)	0.37317 (8)	0.0247 (4)	
H42	0.504288	0.443490	0.366117	0.030*	
C14	0.19709 (8)	0.13711 (17)	0.04254 (8)	0.0306 (4)	
H14	0.160962	0.134436	0.014936	0.037*	
C19	0.32623 (8)	0.47828 (16)	0.23208 (9)	0.0322 (4)	
H19	0.330884	0.406488	0.242582	0.039*	
C17	0.27158 (8)	0.42904 (15)	0.12683 (8)	0.0274 (4)	
C20	0.34444 (8)	0.55349 (16)	0.27455 (9)	0.0322 (4)	
H20	0.361704	0.533286	0.314336	0.039*	
C22	0.35155 (8)	0.74137 (17)	0.30306 (9)	0.0322 (4)	
H22	0.367841	0.725187	0.343748	0.039*	
C23	0.34113 (9)	0.84233 (17)	0.28562 (9)	0.0355 (4)	
H23	0.349714	0.897659	0.313930	0.043*	
C37	0.50447 (9)	0.90062 (15)	0.37861 (9)	0.0325 (4)	
H37	0.500383	0.972306	0.388010	0.039*	
H3	0.4477 (10)	0.3583 (18)	0.4048 (11)	0.037 (6)*	
H4	0.3277 (12)	0.325 (2)	0.1580 (12)	0.050 (7)*	

### Chlorido[5,10,15,20-tetrakis(quinoline-7-carboxamido)porphinato]iron(III) Atomic displacement parameters (Å^2^)

**Table:**

	*U*^11^	*U*^22^	*U*^33^	*U*^12^	*U*^13^	*U*^23^
Fe1	0.01562 (15)	0.01438 (16)	0.01258 (15)	0.000	0.00360 (11)	0.000
Cl1	0.0760 (5)	0.0140 (3)	0.0224 (3)	0.000	0.0114 (3)	0.000
O1	0.0304 (6)	0.0229 (6)	0.0211 (6)	0.0027 (5)	0.0099 (5)	−0.0028 (5)
N1	0.0161 (6)	0.0209 (7)	0.0141 (6)	0.0000 (5)	0.0031 (5)	−0.0001 (5)
N3	0.0269 (7)	0.0189 (7)	0.0182 (7)	−0.0011 (6)	0.0106 (6)	−0.0017 (5)
N2	0.0148 (6)	0.0159 (6)	0.0149 (6)	−0.0024 (5)	0.0042 (5)	−0.0013 (5)
O2	0.0380 (8)	0.0403 (8)	0.0345 (8)	0.0130 (6)	−0.0093 (6)	0.0028 (6)
N6	0.0326 (8)	0.0328 (9)	0.0283 (8)	0.0036 (7)	0.0114 (6)	0.0024 (7)
N5	0.0375 (9)	0.0183 (7)	0.0306 (8)	−0.0004 (6)	0.0126 (7)	−0.0022 (6)
N4	0.0179 (7)	0.0311 (8)	0.0327 (8)	0.0035 (6)	−0.0044 (6)	0.0022 (7)
C5	0.0194 (7)	0.0137 (7)	0.0161 (7)	−0.0019 (6)	0.0060 (6)	−0.0007 (6)
C27	0.0157 (7)	0.0216 (8)	0.0140 (7)	−0.0010 (6)	0.0033 (6)	−0.0005 (6)
C4	0.0199 (8)	0.0132 (7)	0.0145 (7)	−0.0017 (6)	0.0046 (6)	−0.0003 (5)
C33	0.0189 (7)	0.0204 (8)	0.0170 (7)	0.0023 (6)	0.0022 (6)	−0.0010 (6)
C3	0.0221 (8)	0.0181 (8)	0.0140 (7)	−0.0030 (6)	0.0041 (6)	−0.0015 (6)
C6	0.0169 (7)	0.0193 (8)	0.0170 (7)	−0.0008 (6)	0.0064 (6)	−0.0001 (6)
C9	0.0156 (7)	0.0228 (8)	0.0179 (8)	0.0001 (6)	0.0022 (6)	−0.0003 (6)
C40	0.0192 (8)	0.0228 (8)	0.0181 (8)	−0.0016 (6)	0.0020 (6)	−0.0012 (6)
C32	0.0173 (7)	0.0204 (8)	0.0163 (7)	−0.0002 (6)	0.0038 (6)	0.0006 (6)
C36	0.0216 (8)	0.0186 (8)	0.0186 (8)	0.0000 (6)	0.0020 (6)	−0.0022 (6)
C1	0.0164 (7)	0.0170 (7)	0.0155 (7)	−0.0020 (6)	0.0012 (6)	0.0001 (6)
C10	0.0158 (7)	0.0212 (8)	0.0176 (7)	0.0016 (6)	0.0024 (6)	−0.0002 (6)
C11	0.0160 (7)	0.0325 (9)	0.0144 (7)	−0.0004 (7)	0.0037 (6)	0.0000 (7)
C39	0.0252 (8)	0.0258 (9)	0.0217 (8)	−0.0034 (7)	0.0059 (7)	−0.0012 (7)
C2	0.0209 (8)	0.0223 (8)	0.0152 (7)	−0.0016 (6)	0.0016 (6)	−0.0006 (6)
C35	0.0209 (8)	0.0209 (8)	0.0179 (8)	0.0011 (6)	0.0040 (6)	−0.0026 (6)
C34	0.0201 (8)	0.0199 (8)	0.0171 (7)	−0.0002 (6)	0.0027 (6)	−0.0009 (6)
C28	0.0292 (9)	0.0228 (8)	0.0231 (8)	−0.0067 (7)	0.0105 (7)	−0.0030 (7)
C18	0.0197 (8)	0.0339 (10)	0.0263 (9)	0.0058 (7)	0.0040 (7)	0.0037 (7)
C31	0.0243 (8)	0.0250 (9)	0.0175 (8)	0.0018 (7)	0.0071 (6)	−0.0029 (6)
C13	0.0230 (9)	0.0430 (11)	0.0245 (9)	−0.0058 (8)	0.0047 (7)	−0.0095 (8)
C8	0.0156 (8)	0.0385 (10)	0.0213 (8)	−0.0006 (7)	0.0045 (6)	−0.0024 (7)
C26	0.0218 (8)	0.0347 (10)	0.0215 (8)	0.0073 (7)	0.0067 (7)	0.0050 (7)
C41	0.0308 (9)	0.0234 (9)	0.0241 (9)	−0.0001 (7)	0.0122 (7)	−0.0047 (7)
C25	0.0184 (8)	0.0343 (10)	0.0254 (9)	0.0040 (7)	0.0087 (7)	0.0045 (7)
C12	0.0228 (8)	0.0344 (10)	0.0183 (8)	0.0002 (7)	0.0051 (6)	−0.0046 (7)
C38	0.0354 (10)	0.0227 (9)	0.0292 (9)	−0.0044 (7)	0.0099 (8)	0.0031 (7)
C29	0.0340 (10)	0.0283 (9)	0.0246 (9)	−0.0092 (8)	0.0132 (7)	0.0004 (7)
C30	0.0270 (9)	0.0328 (10)	0.0186 (8)	−0.0013 (7)	0.0112 (7)	−0.0001 (7)
C21	0.0170 (8)	0.0378 (10)	0.0272 (9)	0.0009 (7)	0.0044 (7)	0.0037 (8)
C7	0.0175 (8)	0.0373 (10)	0.0197 (8)	−0.0009 (7)	0.0063 (6)	−0.0026 (7)
C16	0.0177 (8)	0.0340 (10)	0.0202 (8)	−0.0010 (7)	0.0028 (6)	0.0006 (7)
C15	0.0175 (8)	0.0423 (11)	0.0235 (9)	0.0028 (7)	−0.0001 (7)	0.0043 (8)
C24	0.0396 (11)	0.0336 (11)	0.0336 (10)	0.0018 (9)	0.0118 (9)	0.0001 (8)
C42	0.0311 (9)	0.0189 (8)	0.0259 (9)	−0.0002 (7)	0.0100 (7)	−0.0046 (7)
C14	0.0175 (8)	0.0519 (12)	0.0213 (9)	−0.0028 (8)	0.0007 (7)	−0.0021 (8)
C19	0.0284 (9)	0.0325 (10)	0.0331 (10)	0.0049 (8)	−0.0016 (8)	0.0085 (8)
C17	0.0236 (9)	0.0334 (10)	0.0245 (9)	0.0046 (7)	0.0027 (7)	0.0051 (7)
C20	0.0260 (9)	0.0405 (11)	0.0272 (9)	0.0033 (8)	−0.0034 (7)	0.0066 (8)
C22	0.0234 (9)	0.0469 (12)	0.0259 (9)	−0.0007 (8)	0.0037 (7)	0.0011 (8)
C23	0.0343 (10)	0.0416 (11)	0.0318 (10)	−0.0018 (9)	0.0091 (8)	−0.0059 (9)
C37	0.0450 (11)	0.0194 (9)	0.0364 (10)	−0.0020 (8)	0.0159 (9)	−0.0019 (7)

### Chlorido[5,10,15,20-tetrakis(quinoline-7-carboxamido)porphinato]iron(III) Geometric parameters (Å, º)

**Table:**

Fe1—Cl1	2.2042 (7)	C2—H2	0.9500
Fe1—N1^i^	2.0508 (13)	C35—H35	0.9500
Fe1—N1	2.0508 (13)	C35—C34	1.370 (2)
Fe1—N2	2.0581 (13)	C34—C42	1.418 (2)
Fe1—N2^i^	2.0581 (13)	C28—H28	0.9500
O1—C33	1.2212 (19)	C28—C29	1.389 (2)
N1—C6	1.381 (2)	C18—C26	1.373 (3)
N1—C9	1.384 (2)	C18—C19	1.422 (3)
N3—C33	1.362 (2)	C18—C17	1.501 (3)
N3—C32	1.413 (2)	C31—H31	0.9500
N3—H3	0.85 (2)	C31—C30	1.386 (2)
N2—C4	1.3811 (19)	C13—H13	0.9500
N2—C1	1.381 (2)	C13—C12	1.392 (2)
O2—C17	1.220 (2)	C13—C14	1.386 (3)
N6—C25	1.373 (2)	C8—H8	0.9500
N6—C24	1.314 (3)	C8—C7	1.356 (2)
N5—C36	1.367 (2)	C26—H26	0.9500
N5—C37	1.314 (2)	C26—C25	1.409 (3)
N4—C16	1.413 (2)	C41—H41	0.9500
N4—C17	1.365 (2)	C41—C42	1.368 (2)
N4—H4	0.86 (3)	C25—C21	1.423 (2)
C5—C27	1.499 (2)	C12—H12	0.9500
C5—C4	1.396 (2)	C38—H38	0.9500
C5—C6	1.399 (2)	C38—C37	1.411 (3)
C27—C32	1.405 (2)	C29—H29	0.9500
C27—C28	1.387 (2)	C29—C30	1.383 (3)
C4—C3	1.439 (2)	C30—H30	0.9500
C33—C34	1.507 (2)	C21—C20	1.413 (3)
C3—H3A	0.9500	C21—C22	1.414 (3)
C3—C2	1.352 (2)	C7—H7	0.9500
C6—C7	1.437 (2)	C16—C15	1.402 (2)
C9—C10	1.396 (2)	C15—H15	0.9500
C9—C8	1.436 (2)	C15—C14	1.376 (3)
C40—C36	1.421 (2)	C24—H24	0.9500
C40—C39	1.414 (2)	C24—C23	1.411 (3)
C40—C41	1.413 (2)	C42—H42	0.9500
C32—C31	1.403 (2)	C14—H14	0.9500
C36—C35	1.408 (2)	C19—H19	0.9500
C1—C10^i^	1.400 (2)	C19—C20	1.370 (3)
C1—C2	1.442 (2)	C20—H20	0.9500
C10—C11	1.500 (2)	C22—H22	0.9500
C11—C12	1.389 (2)	C22—C23	1.359 (3)
C11—C16	1.404 (2)	C23—H23	0.9500
C39—H39	0.9500	C37—H37	0.9500
C39—C38	1.361 (3)		
			
N1^i^—Fe1—Cl1	102.93 (4)	C27—C28—H28	119.6
N1—Fe1—Cl1	102.93 (4)	C27—C28—C29	120.72 (16)
N1^i^—Fe1—N1	154.14 (8)	C29—C28—H28	119.6
N1—Fe1—N2^i^	87.60 (5)	C26—C18—C19	119.81 (18)
N1^i^—Fe1—N2^i^	87.22 (5)	C26—C18—C17	117.61 (16)
N1^i^—Fe1—N2	87.59 (5)	C19—C18—C17	122.20 (17)
N1—Fe1—N2	87.22 (5)	C32—C31—H31	120.2
N2—Fe1—Cl1	101.67 (4)	C30—C31—C32	119.54 (15)
N2^i^—Fe1—Cl1	101.67 (4)	C30—C31—H31	120.2
N2^i^—Fe1—N2	156.66 (7)	C12—C13—H13	120.5
C6—N1—Fe1	124.61 (10)	C14—C13—H13	120.5
C6—N1—C9	105.79 (13)	C14—C13—C12	119.09 (18)
C9—N1—Fe1	126.62 (10)	C9—C8—H8	126.4
C33—N3—C32	128.91 (14)	C7—C8—C9	107.25 (15)
C33—N3—H3	117.3 (16)	C7—C8—H8	126.4
C32—N3—H3	113.8 (16)	C18—C26—H26	119.6
C4—N2—Fe1	125.25 (10)	C18—C26—C25	120.89 (16)
C1—N2—Fe1	127.49 (10)	C25—C26—H26	119.6
C1—N2—C4	105.54 (12)	C40—C41—H41	119.6
C24—N6—C25	117.01 (17)	C42—C41—C40	120.76 (16)
C37—N5—C36	116.81 (15)	C42—C41—H41	119.6
C16—N4—H4	115.2 (17)	N6—C25—C26	118.31 (16)
C17—N4—C16	128.76 (15)	N6—C25—C21	122.29 (17)
C17—N4—H4	115.8 (17)	C26—C25—C21	119.27 (17)
C4—C5—C27	118.36 (14)	C11—C12—C13	120.82 (17)
C4—C5—C6	124.14 (14)	C11—C12—H12	119.6
C6—C5—C27	117.45 (13)	C13—C12—H12	119.6
C32—C27—C5	121.12 (14)	C39—C38—H38	120.7
C28—C27—C5	119.27 (14)	C39—C38—C37	118.69 (17)
C28—C27—C32	119.60 (14)	C37—C38—H38	120.7
N2—C4—C5	125.24 (14)	C28—C29—H29	120.3
N2—C4—C3	110.24 (13)	C30—C29—C28	119.50 (16)
C5—C4—C3	124.44 (14)	C30—C29—H29	120.3
O1—C33—N3	123.59 (15)	C31—C30—H30	119.5
O1—C33—C34	120.77 (15)	C29—C30—C31	121.07 (15)
N3—C33—C34	115.64 (14)	C29—C30—H30	119.5
C4—C3—H3A	126.5	C20—C21—C25	118.88 (17)
C2—C3—C4	107.03 (14)	C20—C21—C22	123.20 (17)
C2—C3—H3A	126.5	C22—C21—C25	117.86 (18)
N1—C6—C5	125.72 (14)	C6—C7—H7	126.4
N1—C6—C7	109.96 (14)	C8—C7—C6	107.14 (14)
C5—C6—C7	124.31 (14)	C8—C7—H7	126.4
N1—C9—C10	126.12 (14)	C11—C16—N4	117.64 (15)
N1—C9—C8	109.83 (14)	C15—C16—N4	122.84 (17)
C10—C9—C8	123.98 (15)	C15—C16—C11	119.52 (17)
C39—C40—C36	117.80 (15)	C16—C15—H15	120.1
C41—C40—C36	118.65 (15)	C14—C15—C16	119.74 (18)
C41—C40—C39	123.54 (15)	C14—C15—H15	120.1
C27—C32—N3	117.36 (14)	N6—C24—H24	117.6
C31—C32—N3	123.08 (15)	N6—C24—C23	124.8 (2)
C31—C32—C27	119.55 (15)	C23—C24—H24	117.6
N5—C36—C40	122.59 (15)	C34—C42—H42	119.7
N5—C36—C35	118.05 (15)	C41—C42—C34	120.66 (16)
C35—C36—C40	119.35 (15)	C41—C42—H42	119.7
N2—C1—C10^i^	125.27 (14)	C13—C14—H14	119.3
N2—C1—C2	110.02 (13)	C15—C14—C13	121.31 (16)
C10^i^—C1—C2	124.67 (14)	C15—C14—H14	119.3
C9—C10—C1^i^	124.11 (14)	C18—C19—H19	119.9
C9—C10—C11	118.76 (14)	C20—C19—C18	120.23 (18)
C1^i^—C10—C11	117.00 (14)	C20—C19—H19	119.9
C12—C11—C10	120.19 (15)	O2—C17—N4	123.52 (18)
C12—C11—C16	119.47 (15)	O2—C17—C18	121.18 (17)
C16—C11—C10	120.31 (15)	N4—C17—C18	115.26 (15)
C40—C39—H39	120.4	C21—C20—H20	119.6
C38—C39—C40	119.10 (16)	C19—C20—C21	120.86 (17)
C38—C39—H39	120.4	C19—C20—H20	119.6
C3—C2—C1	107.16 (14)	C21—C22—H22	120.4
C3—C2—H2	126.4	C23—C22—C21	119.26 (18)
C1—C2—H2	126.4	C23—C22—H22	120.4
C36—C35—H35	119.4	C24—C23—H23	120.6
C34—C35—C36	121.12 (15)	C22—C23—C24	118.78 (19)
C34—C35—H35	119.4	C22—C23—H23	120.6
C35—C34—C33	116.39 (14)	N5—C37—C38	124.99 (17)
C35—C34—C42	119.43 (15)	N5—C37—H37	117.5
C42—C34—C33	124.17 (15)	C38—C37—H37	117.5
			
Fe1—N1—C6—C5	−19.5 (2)	C32—C31—C30—C29	0.0 (3)
Fe1—N1—C6—C7	161.64 (11)	C36—N5—C37—C38	1.1 (3)
Fe1—N1—C9—C10	14.7 (2)	C36—C40—C39—C38	0.5 (2)
Fe1—N1—C9—C8	−162.24 (12)	C36—C40—C41—C42	0.6 (3)
Fe1—N2—C4—C5	16.4 (2)	C36—C35—C34—C33	−179.90 (14)
Fe1—N2—C4—C3	−166.77 (10)	C36—C35—C34—C42	1.1 (2)
Fe1—N2—C1—C10^i^	−11.3 (2)	C1—N2—C4—C5	−177.68 (15)
Fe1—N2—C1—C2	166.51 (11)	C1—N2—C4—C3	−0.84 (17)
O1—C33—C34—C35	−12.0 (2)	C1^i^—C10—C11—C12	84.9 (2)
O1—C33—C34—C42	166.96 (16)	C1^i^—C10—C11—C16	−93.30 (19)
N1—C6—C7—C8	1.0 (2)	C10—C9—C8—C7	−175.04 (17)
N1—C9—C10—C1^i^	−3.2 (3)	C10^i^—C1—C2—C3	177.04 (15)
N1—C9—C10—C11	−179.00 (15)	C10—C11—C12—C13	−176.68 (15)
N1—C9—C8—C7	1.9 (2)	C10—C11—C16—N4	−5.5 (2)
N3—C33—C34—C35	167.42 (15)	C10—C11—C16—C15	175.72 (15)
N3—C33—C34—C42	−13.6 (2)	C11—C16—C15—C14	1.4 (3)
N3—C32—C31—C30	178.28 (15)	C39—C40—C36—N5	−0.2 (2)
N2—C4—C3—C2	0.36 (18)	C39—C40—C36—C35	179.16 (15)
N2—C1—C2—C3	−0.80 (18)	C39—C40—C41—C42	−179.88 (17)
N6—C25—C21—C20	177.71 (17)	C39—C38—C37—N5	−0.8 (3)
N6—C25—C21—C22	0.7 (3)	C35—C34—C42—C41	−1.8 (3)
N6—C24—C23—C22	−0.1 (3)	C28—C27—C32—N3	−178.40 (15)
N5—C36—C35—C34	179.82 (16)	C28—C27—C32—C31	0.9 (2)
N4—C16—C15—C14	−177.35 (16)	C28—C29—C30—C31	1.1 (3)
C5—C27—C32—N3	0.2 (2)	C18—C26—C25—N6	−175.69 (16)
C5—C27—C32—C31	179.55 (14)	C18—C26—C25—C21	0.3 (2)
C5—C27—C28—C29	−178.49 (16)	C18—C19—C20—C21	0.1 (3)
C5—C4—C3—C2	177.23 (15)	C8—C9—C10—C1^i^	173.32 (16)
C5—C6—C7—C8	−177.82 (16)	C8—C9—C10—C11	−2.5 (3)
C27—C5—C4—N2	−178.50 (14)	C26—C18—C19—C20	2.1 (3)
C27—C5—C4—C3	5.1 (2)	C26—C18—C17—O2	−27.1 (3)
C27—C5—C6—N1	−179.83 (14)	C26—C18—C17—N4	155.05 (17)
C27—C5—C6—C7	−1.2 (2)	C26—C25—C21—C20	1.9 (2)
C27—C32—C31—C30	−1.0 (2)	C26—C25—C21—C22	−175.12 (16)
C27—C28—C29—C30	−1.2 (3)	C41—C40—C36—N5	179.38 (16)
C4—N2—C1—C10^i^	−176.82 (15)	C41—C40—C36—C35	−1.3 (2)
C4—N2—C1—C2	0.99 (17)	C41—C40—C39—C38	−179.03 (17)
C4—C5—C27—C32	79.19 (19)	C25—N6—C24—C23	0.9 (3)
C4—C5—C27—C28	−102.17 (18)	C25—C21—C20—C19	−2.1 (3)
C4—C5—C6—N1	−2.7 (3)	C25—C21—C22—C23	0.2 (3)
C4—C5—C6—C7	176.00 (16)	C12—C11—C16—N4	176.33 (15)
C4—C3—C2—C1	0.25 (18)	C12—C11—C16—C15	−2.4 (2)
C33—N3—C32—C27	−179.50 (15)	C12—C13—C14—C15	−1.7 (3)
C33—N3—C32—C31	1.2 (3)	C21—C22—C23—C24	−0.5 (3)
C33—C34—C42—C41	179.28 (16)	C16—N4—C17—O2	−11.7 (3)
C6—N1—C9—C10	175.64 (16)	C16—N4—C17—C18	166.07 (17)
C6—N1—C9—C8	−1.26 (18)	C16—C11—C12—C13	1.5 (2)
C6—C5—C27—C32	−103.49 (18)	C16—C15—C14—C13	0.7 (3)
C6—C5—C27—C28	75.2 (2)	C24—N6—C25—C26	174.62 (16)
C6—C5—C4—N2	4.4 (2)	C24—N6—C25—C21	−1.2 (3)
C6—C5—C4—C3	−172.04 (15)	C14—C13—C12—C11	0.6 (3)
C9—N1—C6—C5	179.00 (15)	C19—C18—C26—C25	−2.3 (3)
C9—N1—C6—C7	0.17 (18)	C19—C18—C17—O2	145.79 (19)
C9—C10—C11—C12	−99.02 (19)	C19—C18—C17—N4	−32.1 (2)
C9—C10—C11—C16	82.8 (2)	C17—N4—C16—C11	−176.58 (17)
C9—C8—C7—C6	−1.8 (2)	C17—N4—C16—C15	2.1 (3)
C40—C36—C35—C34	0.4 (2)	C17—C18—C26—C25	170.75 (15)
C40—C39—C38—C37	−0.1 (3)	C17—C18—C19—C20	−170.57 (17)
C40—C41—C42—C34	1.0 (3)	C20—C21—C22—C23	−176.70 (18)
C32—N3—C33—O1	0.4 (3)	C22—C21—C20—C19	174.78 (18)
C32—N3—C33—C34	−179.03 (15)	C37—N5—C36—C40	−0.6 (3)
C32—C27—C28—C29	0.2 (3)	C37—N5—C36—C35	−179.97 (17)

Symmetry code: (i) −*x*+1, *y*, −*z*+1/2.

### Chlorido[5,10,15,20-tetrakis(quinoline-7-carboxamido)porphinato]iron(III) Hydrogen-bond geometry (Å, º)

**Table:**

*D*—H···*A*	*D*—H	H···*A*	*D*···*A*	*D*—H···*A*
C15—H15···O2	0.95	2.25	2.871 (3)	122
C31—H31···O1	0.95	2.25	2.867 (2)	122
C42—H42···Cl1	0.95	2.77	3.3596 (18)	121
C3—H3*A*···N5^ii^	0.95	2.48	3.411 (2)	166
C14—H14···O1^iii^	0.95	2.45	3.165 (2)	132

Symmetry codes: (ii) −*x*+1, −*y*+1, −*z*+1; (iii) −*x*+1/2, *y*−1/2, −*z*+1/2.
